# Infantile 22q11.2 deletion syndrome with secondary monogenic variants following inconclusive whole exome sequencing: a case report

**DOI:** 10.3389/fped.2026.1964147

**Published:** 2026-09-23

**Authors:** Amir Abadi, Usra I. Ghanem, Bissan Badran, Mohammed Abdallah, Aseel Ba'ba', Fida Al Hijawi, Muna Sharaf, Rami Abu Hajji

**Affiliations:** 1 Faculty of Medicine and Allied Health Sciences, An-Najah National University, Nablus, Palestine; 2 Faculty of Medicine, Al-Quds University, Jerusalem, Palestine; 3 Pediatric Department, Ibn Sina Specialized Hospital, Jenin, Palestine; 4 Primary Health Center, Ministry of Health, Jenin, Palestine; 5 Department of Pediatric Endocrinology, Faculty of Medicine, An-Najah National University, Nablus, Palestine

**Keywords:** 22q11.2 deletion syndrome, blended phenotypes, consanguinity, copy-number variants (CNVS), DiGeorge syndrome, fluorescence *in situ* hybridization, hypoparathyroidism, whole exome sequencing (WES)

## Abstract

**Background:**

Chromosome 22q11.2 deletion syndrome (22q11.2DS), historically known as DiGeorge syndrome, is a common microdeletion disorder characterized by a wide range of multisystem manifestations. While Whole Exome Sequencing (WES) is increasingly used as a first-tier diagnostic tool for complex infant presentations, it has limited sensitivity for detecting copy-number variants such as microdeletions. Parental consanguinity adds further diagnostic complexity by increasing the likelihood of co-occurring single-gene variants.

**Case presentation:**

We present the case of a 12-month-old female born to first-cousin consanguineous parents who presented in early infancy with feeding difficulties, severe growth failure, conotruncal/septal cardiac anomalies (atrial septal defect, pulmonary stenosis, depressed ejection fraction), severe primary hypoparathyroidism with hypocalcemic tetany, and recurrent sepsis. Initial diagnostic WES identified single-nucleotide variants in cardiomyopathy-associated genes (TTN and NEXN) alongside an incidental pathogenic variant in ANO5, but failed to detect a microdeletion. A subsequent, clinically directed Fluorescence *In Situ* Hybridization (FISH) study using the 22q11 “N25” probe established the definitive diagnosis of 22q11.2 microdeletion. Multidisciplinary management including enteral calcium/vitamin D supplementation, heart failure pharmacotherapy, infection prophylaxis, and deferral of live vaccines resulted in clinical and biochemical stabilization.

**Discussion:**

This case highlights the classic expanded phenotype of 22q11.2DS in infancy and demonstrates the technical limitations of WES in identifying microdeletions. It also illustrates how parental consanguinity can introduce overlapping or incidental genetic findings (TTN, NEXN, ANO5) that require careful clinical correlation.

**Conclusion:**

Clinicians must maintain a high index of suspicion for 22q11.2DS based on core clinical features (hypocalcemia, cardiac defects, dysmorphism), as a non-diagnostic or partially explanatory WES result does not rule out microdeletion syndromes. Targeted cytogenetic testing (e.g., FISH or chromosomal microarray) remains essential when microdeletion syndromes are clinically suspected.

## Introduction

Chromosome 22q11.2 deletion syndrome (22q11.2DS), historically known as DiGeorge syndrome (velocardiofacial syndrome), is the most common microdeletion disorder in humans, with an estimated incidence of roughly one in 4,000 live births (1, 2). Chromosome 22q11.2 deletion syndrome (22q11.2DS) encompasses several conditions historically described under distinct clinical names, including DiGeorge syndrome, velocardiofacial syndrome, Shprintzen syndrome, Takao syndrome, and the acronym CATCH 22. Today, 22q11.2 deletion syndrome is the preferred contemporary unifying term to reflect the underlying molecular etiology across this phenotypically heterogeneous spectrum (1). The syndrome arises from a heterozygous, usually *de novo* (approximately 90% of cases), 1.5–3 Mb deletion on the long arm of chromosome 22 that removes dozens of contiguous genes, most notably TBX1, whose haploinsufficiency is thought to underlie much of the pharyngeal-arch phenotype (2, 3). Because the deleted region governs development of structures derived from the third and fourth pharyngeal pouches, affected infants classically present with a triad of conotruncal cardiac malformation, hypoplastic thymus with variable T-cell deficiency, and hypocalcemia secondary to parathyroid hypoplasia (3). While historically characterized by the classic triad of congenital conotruncal heart defects, hypoparathyroidism with hypocalcemia, and thymic hypoplasia leading to immunodeficiency, 22q11.2DS is now recognized as a highly variable multisystem disorder. Its phenotypic spectrum spans neurodevelopmental delays, psychiatric manifestations (such as schizophrenia and anxiety disorders), palatal and craniofacial anomalies, endocrine disturbances, renal malformations, and gastrointestinal dysmotility (3).

Diagnosis of 22q11.2DS in the neonatal or infant period is frequently delayed because no single feature is pathognomonic, and mild or atypical presentations are common (4). Historically, fluorescence *in situ* hybridization (FISH) using probes targeting the deleted region (such as the “N25” probe) served as the diagnostic gold standard, and it remains a rapid, targeted confirmatory test once the syndrome is clinically suspected (5). Chromosomal microarray and multiplex ligation-dependent probe amplification have since become preferred first-tier tests for detecting copy-number variants of this kind (6). A growing body of evidence, however, shows that whole exome sequencing (WES) is now widely used as a first diagnostic step for children with multisystem disease, and has substantially reduced sensitivity for detecting deletions and duplications compared with single-nucleotide variants, because exome capture targets only protein-coding regions non-contiguously, read-depth-based copy-number variant (CNV) remains technically challenging without dedicated, off-target coverage pipelines; microdeletions such as 22q11.2 are a well-recognized example of a diagnosis that can be missed by WES alone (7). This diagnostic gap has practical consequences; infants who undergo exome sequencing for a suspected monogenic cardiomyopathy or metabolic disorder may still require a dedicated cytogenetic test if their broader phenotype (dysmorphism, hypocalcemia, feeding difficulty) suggests a microdeletion syndrome.

A further layer of diagnostic complexity arises in the setting of parental consanguinity, which increases the prior probability of multiple co-occurring single-gene (usually recessive) conditions in the same child, sometimes producing a composite or blended phenotype, and increases the yield of incidental or secondary findings unrelated to the presenting illness (8–10). Exome-based cohorts suggest that more than one clinically relevant molecular finding is identified in roughly 2%–7.5% of diagnosed patients overall, with a two-to three fold higher frequency among children of consanguineous parents (9, 11).

In this report, we present a case of a female infant, born to first-cousin parents, who presented in the early neonatal period with feeding difficulty, a cardiac murmur, and subsequently severe hypocalcemic tetany, recurrent sepsis, and growth failure. Diagnostic whole exome sequencing identified variants in two cardiomyopathy-associated genes (TTN and NEXN) together with an incidental pathogenic variant in ANO5, but only a subsequent, clinically directed FISH study established the underlying diagnosis of 22q11.2 microdeletion/DiGeorge syndrome.

## Case presentation

The patient is a 12-month-old female born at 36 weeks gestational age via emergent Cesarean section due to severe maternal preeclampsia. Her birth weight was 2.3 kgs (<10th percentile, small for gestational age). Neonatal follow-up was regular with a gynecologist, and a detailed ultrasound was done and was normal at the time. The patient's family history is significant for parental consanguinity (first cousins).

Clinical concerns began in the early neonatal period, with persistent nasal congestion, feeding fatigue with profuse diaphoresis during bottle-feeding, sub-optimal weight gain, and a prominent heart murmur detected during initial outpatient evaluation. Over time, these symptoms evolved into overt cardiovascular insufficiency, recurrent neonatal apnea, severe hypocalcemic tetany, choking spells with oral/nasal regurgitation during feeding, and localized cutaneous necrosis. The patient experienced multiple complex hospitalizations during her first year of life (summarized chronologically in Table 1).

**Table 1:** Chronological summary of hospitalizations.

Age	Episode of care and clinical milestone	Diagnostic and therapeutic events
2–3 weeks	Outpatient Presentation	Diaphoresis during feeding, poor weight gain, and persistent heart murmur noted.
1 month	First NICU Admission	Initial echocardiogram revealed LV systolic dysfunction (EF = 43%), ASD, and PS. Initiated on Captopril, Lasix, and Carvedilol. Severe hypocalcemia (Ca = 6.2 mg/dL) and primary hypoparathyroidism (PTH = 18.5 pg/mL) identified; oral calcium added. Hypotonia noted. Transferred to a local governmental hospital.
1 month	Critical Sepsis & Deterioration	Developed high fever (39.1 °C), high inflammation (CRP = 153 mg/L), severe apnea, and septic shock at local governmental hospital requiring intubation.
1 month	Second NICU Admission	Readmitted intubated; extubated to room air within 12 h. Severe calcium nadir (5.3 mg/dL) treated with IV calcium, oral Alfacalcidol, and oral Magnesium. Blood cultures grew Enterobacter cloacae and urine grew Klebsiella; treated with 14 days of IV Meropenem/Tazocin/Amikacin.
2 months	Third NICU Admission	Admitted for acute choking spell and cyanosis during bottle feeding. Treated with NPO status, temporary OG tube, and 5-day course of IV Cefotaxime/Cloxacillin. Failure to grow was noted. Discharged with fully oral feeding.
4 months	Fourth Hospital Admission	Presenting with fever (39 °C), upper respiratory tract infection/bronchiolitis, and scalp tissue necrosis secondary to IV calcium extravasation. Treated with IV Vancomycin/Ceftriaxone and daily topical MEBO/Fusidic acid wound care.
10 months	Genetic Testing Results (WES)	Whole Exome Sequencing identified TTN likely pathogenic variant, NEXN VUS, and ANO5 pathogenic incidental variant.
12 months	Definitive Genetic Diagnosis	Confirmatory FISH analysis demonstrated 22q11.2 microdeletion.

At 12 months of age, physical examination revealed severe failure to thrive, weighing 6.5 kgs (below the 3rd percentile) with a body length of 67 cm (3rd percentile) and a head circumference of 44 cm (<−2 standard deviations). Detailed dysmorphic evaluation demonstrated craniofacial features characteristic of 22q11.2 microdeletion, including low-set, posteriorly rotated ears with thick helices, a long smooth philtrum, retrognathia, a thin upper lip, and a bulbous nasal tip. Cardiovascular evaluation revealed a persistent Grade II/VI systolic ejection murmur loudest along the left upper sternal border, without evidence of active pulmonary congestion or peripheral edema under pharmacologic maintenance. The abdominal examination demonstrated mild distension with a small, reducible umbilical hernia and absence of hepatosplenomegaly. Skin examination was notable for scarring secondary to past peripheral intravenous calcium gluconate extravasation on the distal lower extremity and an area of healing, re-epithelialized tissue over the scalp. Neurologically, the infant exhibited marked axial hypotonia, late achievement of unsupported sitting at 9.5 months, delayed fine motor skills characterized by an immature pincer grasp, and delayed vocalization consisting primarily of bilabial babbling without formal word formation.

The diagnostic approach integrated serial physical examinations, targeted laboratory testing, cardiovascular and renal imaging, and advanced cytogenetic modalities [Whole Exome Sequencing (WES) and targeted Fluorescence *In Situ* Hybridization (FISH)]. Initial laboratory evaluations confirmed severe hypocalcemia (Total Ca = 5.3–6.2 mg/dL, Ionized Ca = 0.80 mmol/L) accompanied by hyperphosphatemia (10.4 mg/dL), hypomagnesemia (1.39 mg/dL), and inappropriately low parathyroid hormone levels (18.5 pg/mL), confirming primary hypoparathyroidism. Immunological investigation was to evaluate T-cell subsets (CD3 = 1,100 cells/µL, CD4 = 800 cells/µL, CD8 = 650 cells/µL) given the suspected thymic hypoplasia.

Echocardiography demonstrated non-conotruncal cardiac defects, specifically a secundum atrial septal defect (ASD) and peripheral pulmonary artery stenosis. While classic conotruncal malformations (such as tetralogy of Fallot, truncus arteriosus, and interrupted aortic arch) predominate in 22q11.2DS, non-conotruncal anomalies including isolated septal defects and branch pulmonary artery stenosis are well-recognized components of the broader cardiovascular spectrum associated with the syndrome.

Neuroimaging via brain MRI was done to investigate global developmental delay and demonstrated prominent frontal subarachnoid spaces and a cavum septum pellucidum. Renal and abdominal ultrasounds ruled out nephrocalcinosis, demonstrating only mild left renal pelvic fullness and mild parenchymal echogenicity.

Comprehensive genetic testing via Whole Exome Sequencing (WES) at 10 months of age revealed two heterozygous variants associated with cardiomyopathy: a pathogenic nonsense variant in the TTN gene NM_001267550.2: exon 41:c.9577C > T, p.Arg3193*; ACMG criteria PVS1, PM2) associated with autosomal dominant dilated cardiomyopathy (OMIM #604145) and a variant of uncertain significance (VUS) in the NEXN gene (NM_144573.3: exon 12: c.1582_1584del,p.Glu528del; ACMG criteria PVS1, PM2), associated with autosomal dominant dilated/hypertrophic cardiomyopathy (OMIM #613122). In addition to an incidental pathogenic heterozygous nonsense variant in the ANO5 NM_213599.2: exon 11: chr11:22272359 T > A; mean coverage depth 325X; c.1086T > A, p.Tyr362*; ACMG criteria PVS1, PM2, PP5), associated with autosomal dominant gnathodiaphyseal dysplasia (OMIM#166260).

Because the cardiac defects (ASD, PS) and systemic manifestations (severe hypocalcemia, primary hypoparathyroidism, dysmorphism) were not fully explained by these single-gene findings, a subsequent targeted Fluorescence *In Situ* Hybridization (FISH) study utilizing the 22q11 “N25” probe was performed, establishing the definitive underlying diagnosis of heterozygous 22q11.2 microdeletion. Genetic results can be viewed in Figures 1, 2.

**Figure 1:**
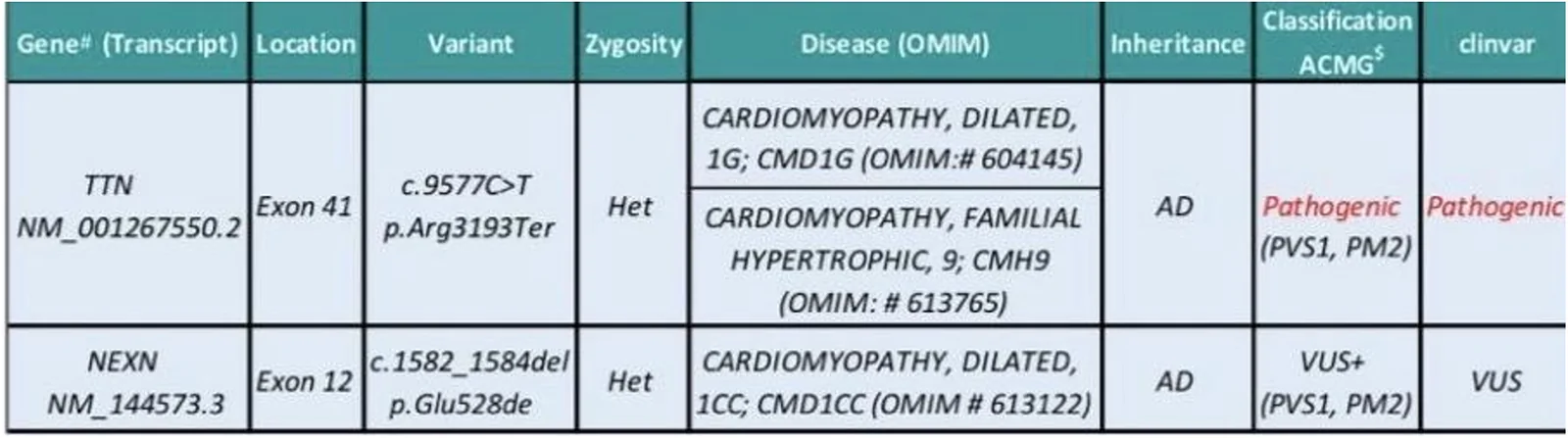
Whole exome sequencing (WES) results of two heterozygous mutations in TTN and NEXN genes.

**Figure 2:**
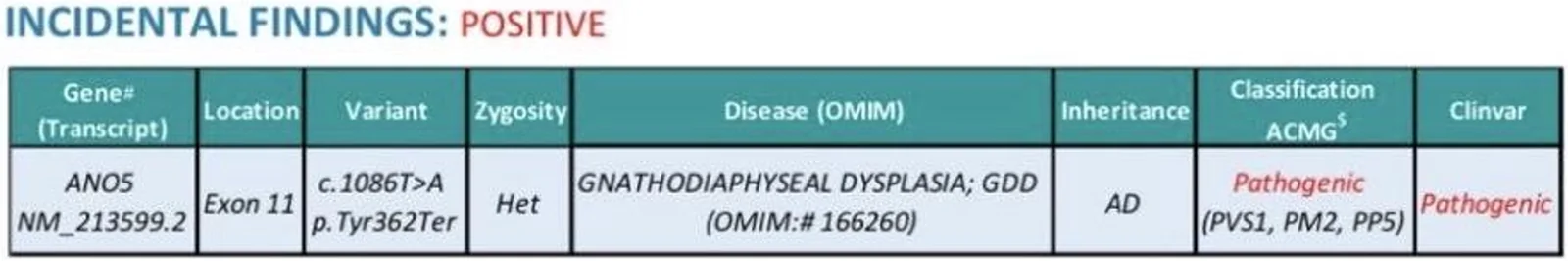
Whole exome sequencing (WES) results incidental finding of heterozygous mutation of ANO5 gene.

The primary diagnosis was established as 22q11.2 Microdeletion/DiGeorge Syndrome, complicated by primary hypoparathyroidism, congenital heart disease, severe growth failure, and global developmental delay. Alternative differential diagnoses considered during her course included isolated familial dilated cardiomyopathy (given the TTN and NEXN variants), isolated idiopathic hypoparathyroidism, and autosomal dominant craniometaphyseal dysplasia (due to the ANO5 variant). Prognostically, her outcome depends on maintaining strict normocalcemia to prevent seizures or nephrocalcinosis, ongoing response to anti-heart failure pharmacotherapy, and close developmental and immunological surveillance.

Heart failure was managed with Captopril (0.1 mg PO q8 h), Furosemide (2 mg PO q12 h), and Carvedilol (0.2 mg PO q12 h). Severe hypoparathyroidism was stabilized using enteral Calcium Carbonate (400 mg PO q6 h; 72 mg/kg/day elemental calcium), Alfacalcidol (0.25 mcg PO q12 h), and oral Magnesium Sulfate (270 mg PO q6 h). To prevent extravasation tissue injury, IV calcium was transitioned to oral maintenance as soon as biochemically feasible. Due to suspected immune deficiency, live attenuated vaccines were withheld pending T-cell quantification, and daily Co-Trimoxazole (0.8 mL PO q24 h) was initiated for infection prophylaxis. Scalp tissue necrosis from IV extravasation was managed with daily topical MEBO and fusidic acid ointment until complete healing.

Follow-up assessments by both clinicians and the patient's family demonstrated stabilization across multiple organ systems following the implementation of targeted therapy. Serum total calcium levels were maintained within acceptable target ranges (7.6–8.8 mg/dL) without recurrent tetany or systemic seizures, and serum magnesium stabilized at 2.07 mg/dL. Serial echocardiography documented stable left ventricular systolic function with an ejection fraction remaining between 43% and 48%, without progression of pulmonary artery stenosis. Renal ultrasound examinations confirmed the absence of nephrocalcinosis or urolithiasis despite prolonged high-dose calcium supplementation.

Long-term cardiovascular management requires a structured, multi-disciplinary approach. Given the co-occurrence of conotruncal/septal defects (ASD, PS) alongside heterozygous variants in cardiomyopathy-associated genes (TTN and NEXN), serial echocardiography is planned every 3–6 months to monitor left ventricular systolic function, ejection fraction, and right ventricular pressure gradients. Pharmacotherapy with afterload reduction (Captopril) and beta-blockade (Carvedilol) will be continuously adjusted based on body weight and ventricular remodeling parameters. Diuretic therapy (Furosemide) will be titrated to maintain euvolemia while avoiding intravascular volume depletion. Pediatric cardiology follow-up will also screen for late-onset arrhythmias or progressive outflow tract obstruction.

## Discussion

The clinical course described here aligns with the multisystem phenotype that has been documented in large cohorts of infants with 22q11.2DS. In a series of 87 patients with confirmed 22q11.2 deletion, congenital heart disease was present in 86.2%, failure to thrive in 58.6%, and major infections in 75.9%, with hypoparathyroidism and neuropsychiatric or developmental disorders occurring at significantly higher rates in affected children (12). Our patient's echocardiographic findings of a secundum atrial septal defect with pulmonary artery stenosis and depressed ejection fraction, her severe primary hypoparathyroidism with recurrent tetany, her recurrent bacteremic sepsis, and her marked failure to thrive with axial hypotonia and delayed motor and language milestones together recapitulate this expanded phenotype rather than representing an atypical course.

The constellation of infant feeding difficulty, a cardiac murmur, severe hypocalcemic tetany, recurrent sepsis, and growth failure should raise strong, early clinical suspicion for 22q11.2DS. 22q11.2DS is an extremely heterogeneous multisystem condition with over 100 reported clinical features across cardiac, endocrine, immunological, palatal, neurodevelopmental, and gastrointestinal domains. International consensus guidelines emphasize that early diagnosis allows for proactive, anticipatory surveillance and timely interventions, such as calcium restoration, immune evaluation prior to administering live vaccines, and feeding support, which significantly mitigate pediatric morbidity (13).

While 22q11.2 deletion syndrome is classically associated with congenital heart disease, specifically conotruncal defects such as tetralogy of Fallot or interrupted aortic arch, it does not typically manifest as primary dilated cardiomyopathy (DCM) (14).

Instead, the patient's DCM and severe ventricular dysfunction are far more consistent with the identified pathogenic nonsense variant in TTN. Truncating variants in TTN (TTNtv) are well-established, high-penetrance drivers of DCM. Together, these findings establish a true dual molecular diagnosis: the 22q11.2 deletion drives the extra-cardiac/syndromic phenotype, whereas the TTN truncating variant independently drives the cardiomyopathy. This highlights the critical importance of comprehensive genomic evaluation beyond initial chromosomal microarray, as relying solely on 22q11.2 as an explanation for the cardiac phenotype would have overlooked the primary monogenic etiology.

Genomic sequencing revealed a pathogenic TTN variant, establishing a dual molecular diagnosis. Truncating variants in TTN (TTNtv) are well-established causes of dilated cardiomyopathy and severe cardiac dysfunction. In this patient, the combination of the 22q11.2 deletion and the TTN pathogenic variant demonstrates a dual diagnostic etiology, wherein distinct genetic loci independently or synergistically drive separate components of the overall clinical phenotype.

In contrast, the clinical relevance of the secondary sequence variants remains limited by current evidence:

In addition, a heterozygous variant was identified in NEXN Variant (VUS). NEXN encodes nexilin, an F-actin binding protein crucial for Z-disc stability in cardiomyocytes. Although alterations in NEXN have been implicated in dilated and hypertrophic cardiomyopathies, this identified variant currently meets criteria for a VUS under ACMG/AMP guidelines. It remains plausible that this variant acts as an oligogenic modifier exacerbating the cardiac phenotype, but current evidence cannot establish a direct pathogenic role.

ANO5 mutations are classically associated with autosomal recessive limb-girdle muscular dystrophy type R12 (LGMD2L) and Miyoshi myopathy-3. The patient exhibited no overt proximal or distal muscle weakness, elevated creatine kinase (CK) levels, or skeletal myopathy symptoms. Because ANO5-related disorders are typically recessive and the patient presented without matching neuromuscular features, this variant is interpreted as an incidental VUS without clear phenotypic correlation. However, given the potential for late-onset muscular manifestations, long-term pediatric neurology and neuromuscular longitudinal follow-up is warranted as the patient matures.

The diagnostic sequence in this case is itself instructive. Whole exome sequencing was performed first, given the family history of consanguinity and the cardiac phenotype, and it successfully identified single-nucleotide variants in TTN and NEXN as well as an incidental ANO5 variant, but it did not detect the 22q11.2 microdeletion. This is consistent with the technical literature on WES, which reports sensitivity below roughly 60% for copy-number variants larger than 50 base pairs and specifically cites 22q11.2 microdeletion as a diagnosis that can be missed because of insufficient, discontinuous probe coverage across the deleted interval (7). Multiple independent groups examining WES-based copy-number calling have likewise concluded that, even with dedicated bioinformatic pipelines, exome data cannot yet substitute for a chromosomal microarray or a targeted FISH study when a microdeletion or microduplication syndrome is clinically suspected (7, 15). The eventual use of the 22q11.2 “N25” FISH probe in this case, prompted by her hypocalcemia, cardiac defect, and dysmorphic features, therefore represents the appropriate orthogonal test rather than a redundant one, and this sequence is a pattern clinicians should anticipate whenever a contiguous-gene deletion syndrome remains plausible despite a negative or partially informative exome result.

Current international guidelines recommend chromosomal microarray (CMA) or multiplex ligation-dependent probe amplification (MLPA) as preferred first-tier methodologies for genome-wide copy-number variant (CNV) detection due to their ability to define exact deletion boundaries and detect atypical microdeletions (15). In our clinical setting, targeted Fluorescence *In Situ* Hybridization (FISH) utilizing the 22q11 “N25” probe was selected as the confirmatory diagnostic test primarily due to local resource availability, lower cost, and rapid turnaround time. While FISH is inherently limited to locus-specific hybridization and cannot delineate deletion size or identify concurrent structural variants elsewhere in the genome, it provided a rapid, highly accurate, and cost-effective orthogonal confirmation of the classic 22q11.2 microdeletion once the clinical phenotype (hypocalcemia, cardiac malformation, and subtle dysmorphism) strongly pointed to DiGeorge syndrome.

Furthermore, this case highlights a broader paradigm shift in diagnostic genomics. While hybrid-capture WES provides high depth over targeted exons (e.g., >300X depth in our patient), its non-contiguous nature leaves a well-recognized blind spot for structural CNVs. High-throughput diagnostic workflows are increasingly shifting toward Whole Genome Sequencing (WGS) as a first-tier assay. WGS provides uniform physical coverage across both coding and non-coding regions allowing simultaneous detection of SNVs, indels, and microdeletions in a single test. Where WGS is available, the diagnostic delay and necessity for secondary orthogonal testing experienced in this case can be effectively circumvented.

It is important to clarify that Whole Exome Sequencing (WES) platforms vary significantly in their bioinformatic capabilities. Modern diagnostic exome pipelines increasingly incorporate specialized read-depth copy-number variant (CNV) calling algorithms, off-target reads analysis, or hybrid-capture enrichment designed to detect structural variants. In our patient's case, the standard bioinformatic pipeline utilized for WES was optimized primarily for single-nucleotide variants (SNVs) and small insertions/deletions (indels), and did not include dedicated, validated CNV calling tools. Consequently, the non-detection of the 22q11.2 microdeletion reflects the specific pipeline configuration used rather than an absolute, intrinsic limitation of all contemporary exome sequencing platforms (7).

The finding of concurrent TTN and NEXN variants raises a separate question about how much they contribute independently to this infant's cardiac phenotype. Truncating TTN variants are the single most common monogenic cause of dilated cardiomyopathy, identified in up to a quarter of cases, although penetrance is incomplete and disease expression is influenced by variant location and by additional genetic or environmental modifiers (16, 17). NEXN encodes a Z-disc protein that stabilizes the cardiac sarcomere; pathogenic variants have been linked to both dilated and hypertrophic cardiomyopathy, though the gene–disease association is still considered only moderate and the great majority of reported evidence comes from small series (18). Given that our patient's cardiac defects (ASD, pulmonary stenosis) are conotruncal/septal anomalies typical of 22q11.2DS itself rather than a primary cardiomyopathy, it is plausible that the TTN and NEXN findings represent an incompletely penetrant, co-inherited predisposition to later ventricular dysfunction layered on top of a structural, deletion-driven heart defect, rather than the primary explanation for her presentation. Distinguishing these contributions will require longitudinal echocardiographic follow-up specifically for early ventricular dysfunction, since consanguinity is well recognized to increase the likelihood of more than one clinically relevant genetic finding in a single child, occurring in roughly two to three times as many consanguineous as non-consanguineous families in large exome cohorts (9, 11, 19). The incidentally identified ANO5 variant, associated with gnathodiaphyseal dysplasia, is a further example of this phenomenon: it does not appear to explain any element of the current phenotype but underscores why secondary findings must be interpreted cautiously and correlated with the clinical picture rather than assumed to be causative simply because they are pathogenic.

Beyond classic structural anomalies (ASD, pulmonary stenosis), our patient exhibited depressed left ventricular systolic function (EF 43%–48%). While conotruncal/septal defects are direct manifestations of TBX1 haploinsufficiency in 22q11.2DS, early infant myocardial failure is less typical of isolated microdeletions without severe hemodynamic strain. While early infant myocardial failure is less typical of isolated 22q11.2 microdeletions, attributing this phenotype to a synergistic “dual-hit” mechanism between 22q11.2DS and the TTN/NEXN variants remains speculative. Truncating TTN variants (TTNtv) are well-established drivers of dilated cardiomyopathy, and NEXN plays an important role in sarcomeric stability; thus, these variants may have imparted an underlying myocardial vulnerability that unmasked early cardiac failure under hemodynamic stress. However, in the absence of functional assays or family segregation studies, a definitive oligogenic or synergistic interaction cannot be proven. Importantly, parental genetic testing was not performed in this family. Segregation analysis is essential to determine inheritance patterns, establish cis/trans phasing, and reclassify variants of uncertain significance (VUS). Until such studies or functional validations are conducted, the contribution of the TTN and NEXN findings to the infantile phenotype must be interpreted with caution.

Management of this infant's hypoparathyroidism followed the standard approach for infants under 1 year, in whom conventional therapy combines enteral calcium carbonate with active vitamin D and correction of coexisting hypomagnesemia, since magnesium deficiency itself impairs parathyroid hormone secretion and calcium repletion (20). Current guidelines also emphasizes transitioning from intravenous to oral calcium as rapidly as hemodynamically and biochemically feasible, precisely because peripheral intravenous calcium is a recognized cause of extravasation injury and secondary calcinosis cutis, a complication this infant experienced with scalp tissue necrosis (20, 21). Her subsequent stabilization on oral calcium, alfacalcidol, and magnesium, with serum calcium maintained in an acceptable low-normal range without recurrent seizures, mirrors the intended trajectory of guideline-directed therapy for pediatric hypoparathyroidism.

Growth failure of the severity documented in this infant is also a well-recognized, if under-appreciated, feature of 22q11.2DS. Short stature and postnatal growth failure have been reported even in children without cardiac defects or classic dysmorphism, and isolated growth hormone deficiency has been described in at least one infant with 22q11.2 microdeletion whose growth failure was the presenting feature and who responded to growth hormone therapy (22). This raises a reasonable question for this patient's ongoing care: if nutritional optimization and cardiac stabilization do not correct her growth trajectory, an evaluation of the growth hormone axis would be a logical next step, as has been suggested in similar cases.

The decision to withhold live attenuated vaccines (OPV, rotavirus, MMR) following T-cell subset quantification is consistent with current immunological practice guidelines for 22q11.2DS, which recommend flow cytometric and T-cell functional assessment before administering live vaccines, typically around 9–12 months of age (23, 24). Published safety data indicate that live vaccines are generally well tolerated in children who meet minimum T-cell thresholds (commonly cited as CD3 ≥ 500 cells/µL or CD8 ≥ 200 cells/µL), with adverse events largely confined to children with more profound lymphopenia or complete 22q11.2 deletion syndrome (23, 25). Given this infant's history of recurrent bacterial sepsis with organisms such as Enterobacter cloacae and Klebsiella species, and the broader recognition that thymic hypoplasia predisposes to both cellular and, later, humoral immune deficits, her ongoing Pneumocystis prophylaxis with co-trimoxazole and deferral of live immunization until immune status is clarified represent an appropriately cautious approach pending the results of formal T-cell enumeration.

Comprehensive genetic counseling is essential for the family given the parental consanguinity (first cousins) and the multi-locus genetic findings. Targeted chromosomal microarray or FISH testing should be offered to both parents to determine whether the 22q11.2 microdeletion occurred *de novo* or was inherited from a parent with subtle/variable expression or a balanced chromosomal rearrangement. If the deletion is *de novo*, the recurrence risk for future pregnancies is low (<1%). If either parent carries the microdeletion or a balanced translocation, the recurrence risk rises to 50% for each subsequent pregnancy (25). For future pregnancies, prenatal diagnostic options such as chorionic villus sampling (CVS) or amniocentesis with chromosomal microarray, as well as pre-implantation genetic testing for monogenic/structural variants (PGT-M/SR), should be discussed. Reproductive counseling must also address carrier status for autosomal recessive disorders given the background of parental consanguinity.

A key limitation of this case was the omission of critical diagnostic screening modalities during the initial neonatal period. Pulse oximetry screening for critical congenital heart disease was not performed prior to initial discharge, which contributed to a delayed recognition of the patient's structural cardiac anomalies and underlying genetic diagnosis. Additionally, serum thyroid function testing (TSH and Free T4) was regrettably omitted during her initial hospitalizations, leaving potential thyroid dysgenesis or central hypothyroidism unassessed during her acute evaluation for developmental delay, hypotonia, and growth failure.

A notable limitation of this report is that the precise physical boundaries and megabase extent of the 22q11.2 deletion could not be defined because diagnostic confirmation was established via locus-specific FISH rather than chromosomal microarray (CMA) or multiplex ligation-dependent probe amplification (MLPA). While FISH confirmed the classic deletion using the N25 probe, it cannot distinguish between typical 3 Mb deletions, nested 1.5 Mb deletions, or atypical microdeletions. Consequently, exact genotype–phenotype correlations—such as mapping specific clinical features to particular haploinsufficient genes within the 22q11.2 locus—could not be explored in this patient.

## Conclusion

This case highlights the clinical complexity and phenotypic variability of 22q11.2 deletion syndrome, particularly when compounded by concurrent genetic variations. While the patient's infantile dilated cardiomyopathy and tissue fragility presented atypical features, the identified variants in TTN, NEXN, and ANO5 must be interpreted with caution. These genetic findings are currently considered potentially contributory or incidental variants rather than established phenotypic drivers, as parental segregation and functional characterization were not performed. Furthermore, this case highlights important diagnostic nuances in genomic screening: the failure of initial whole-exome sequencing (WES) to identify the microdeletion highlights limitations inherent to the specific exome capture kit and bioinformatic pipeline utilized, rather than a universal constraint of all WES platforms, many of which now incorporate robust CNV-calling algorithms. When clinical suspicion for 22q11.2DS remains high despite negative or inconclusive WES findings, orthogonal testing such as chromosomal microarray or FISH remains essential for definitive diagnosis and appropriate management.

## Data Availability

The genetic sequence data supporting the findings of this case report are not publicly available due to patient privacy and ethical constraints. De-identified data are available from the corresponding author upon reasonable request.
